# Soda and Sports Drink Consumption and Life Stressors Among High School Students: Youth Risk Behavior Survey, 2023

**DOI:** 10.1111/josh.70201

**Published:** 2026-08-04

**Authors:** Ann M. Goding Sauer, Ryan Saelee, Samantha L. Pierce, Caitlin L. Merlo, Jessica R. Hunter, Julie L. Self

**Affiliations:** ^1^ Division of Nutrition Physical Activity and Obesity (DNPAO) National Center for Chronic Disease Prevention and Health Promotion (NCCDPHP), Centers for Disease Control and Prevention (CDC) Atlanta Georgia USA; ^2^ Division of Adolescent and School Health NCCDPHP, CDC Atlanta Georgia USA; ^3^ McKing Consulting Corporation, DNPAO NCCDPHP, CDC Atlanta Georgia USA

**Keywords:** adolescents, chronic disease, health behaviors, life stressors, soda, sugar‐sweetened beverages

## Abstract

**Background:**

Life stressors might influence health behaviors, including frequent sugar‐sweetened beverage (SSB) consumption. We described associations between life stressors and soda/sports drink (SD) consumption among US high school students.

**Methods:**

We tested associations between life stressors and daily (≥ 1 time/day) and heavy daily (≥ 3 time/day) consumption of soda/SD using the 2023 national Youth Risk Behavior Survey (daily *n* = 12,308; heavy daily *n* = 11,980). We calculated prevalence ratios (aPRs) and 95% confidence intervals (CI) adjusted for grade, sex, weight perception, and race and ethnicity. Not experiencing the stressor was the reference group.

**Results:**

Daily consumption was significantly higher among those who experienced unstable housing (aPR = 1.6, 95% CI = 1.2, 2.2), lacking a household adult trying to meet basic needs (aPR = 1.3, 95% CI = 1.1, 1.5), bullying at school (aPR = 1.3, 95% CI = 1.2, 1.4), missing school due to safety concerns (aPR = 1.3, 95% CI = 1.1, 1.5), or feeling unfairly disciplined at school (aPR = 1.2, 95% CI = 1.1, 1.3). Results for heavy daily consumption were similar.

**Implications for School Health Policy, Practice and Equity:**

Initiatives to reduce soda/SD consumption could benefit students with relatively high consumption prevalence, including those experiencing life stressors.

**Conclusions:**

The prevalence of consuming soda/SD was higher among US high school students who experienced life stressors than those who did not.

## 1 Introduction

Assessing dietary behaviors—including consumption of sugar‐sweetened beverages (SSBs) such as soda/pop (soda) and sports drinks—among adolescents is important given the long‐lasting and far‐reaching health effects of nutrition during this phase of life [1]. Furthermore, the Dietary Guidelines for Americans 2020–2025 recommend limited intake (< 10% of total daily calories) of added sugars [2]. Despite recommendations, during 2015–2018, 41% of youth aged 12–19 years consumed more than 15% of their total calorie intake in added sugars [3]; SSBs were a major source [3, 4]. Frequent consumption of SSBs has been associated with unhealthy weight gain, type 2 diabetes, cardiovascular disease risk, and cavities [5, 6, 7, 8]. In addition, socioeconomic factors, health behaviors, and the physical environment contribute to health outcomes [1].

In 2023, 15% of US high school students drank sugar‐sweetened soda daily, and 11% drank sports drink daily [9] with variations by grade [9], sex, and race and ethnicity [9, 10]. Although the overall prevalence of daily consumption has decreased (soda: 34% in 2007, sports drink: 14% in 2015) [9], frequent consumption of these beverages continues to jeopardize high school students' health. For example, recent national data show that, among those aged 12–19 years, 22% have obesity and 56% have cavities [11].

Furthermore, evidence suggests adolescents might employ unhealthy behaviors as a way of coping with life stressors [12]. The developing adolescent brain is particularly susceptible to the effects of stress [13, 14, 15], and stress during adolescence has been associated with unhealthy eating behaviors [16, 17]. More specifically, experiences of bullying or other violence at school have been associated with soda consumption [18]. Also, adolescents who experienced housing instability have an increased risk for unhealthy behaviors such as substance abuse and risky sexual health behaviors [19]. Better understanding the relationship between life stressors and unhealthy behaviors—such as soda and sports drink consumption—among adolescents could help identify potential strategies to improve their diet and health.

The objective of this study is to describe the association between soda and sports drink consumption—the two types of SSBs included in the national Youth Risk Behavior Survey (YRBS)—and household stressors and school stressors among US high school students in 2023.

## 2 Methods

### 2.1 Participants

We used data from the national YRBS, which is a school‐based cross‐sectional survey that collects data regarding key health‐related behaviors [20]. The data are nationally‐representative of US high school (grades 9–12) students attending public and private schools. The YRBS data are anonymous, publicly available, and used for surveillance purposes; this study was exempt from Institutional Review Board review. The 2023 national YRBS includes 20,103 usable questionnaires; response rates and other details are available elsewhere [20].

### 2.2 Instrumentation

Table 1 reflects the survey questions and analytic coding for our measures. The outcome variables were derived from two survey questions, one pertaining to soda consumption (excluding diet soda/pop) and the other pertaining to sports drink consumption (excluding low‐calorie sports drinks). The two combined outcome variables were defined by first calculating daily consumption for each beverage type, summing across the two variables, and then categorizing respondents based on consumption frequency (daily: consumed soda or sports drink ≥ 1 time per day;heavy daily: consumed soda or sports drink ≥ 3 times per day), which is consistent with methods published elsewhere [21]. If data for either soda or sports drink were missing but enough information was available on the nonmissing drink to categorize the respondent as consuming soda or sports drink at least once per day (for daily consumption) or at least three times per day (for heavy daily consumption), the respondent was included. However, if data were missing for both soda and sports drink or otherwise not enough information was available to categorize, the respondent was excluded. To better understand the implications of this outcome definition we conducted a sensitivity analysis with a more restrictive definition, excluding all observations missing data for either soda or sports drink consumption.

**TABLE 1:** Question wording and analytic coding for independent and dependent variables—Youth Risk Behavior Survey, High School Students, United States, 2023.

Variable	Questionnaire wording	Questionnaire answerresponses	Analytic coding
Outcome of interest			
Soda/sports drink consumption	During the past 7 days, how many times did you drink a can, bottle, or glass of soda or pop, such as Coke, Pepsi, or Sprite? (Do not include diet soda or diet pop.)	I did not drink soda or pop during the past 7 days	0 times per day
		1–3 times during the past 7 days	0.286 times per day
		4–6 times during the past 7 days	0.714 times per day
		1 time per day	1 time per day
		2 times per day	2 times per day
		3 times per day	3 times per day
		4 or more times per day	4 or more times per day
		Missing	—
	During the past 7 days, how many times did you drink a can, bottle, or glass of a sports drink such as Gatorade or PowerAde? (Do not count low‐calorie sports drinks such as Propel or G2.)	I did not drink sports drinks during the past 7 days	0 times per day
		1–3 times during the past 7 days	0.286 times per day
		4–6 times during the past 7 days	0.714 times per day
		1 time per day	1 time per day
		2 times per day	2 times per day
		3 times per day	3 times per day
		4 or more times per day	4 or more times per day
		Missing	—
Student characteristics			
School grade level	In what grade are you?	9th	9th
		10th	10th
		11th	11th
		12th	12th
		Ungraded or other grade	—
		Missing	—
Sex	What is your sex?	Female	Female
		Male	Male
		Missing	—
Race and ethnicity	Are you Hispanic or Latino?	Yes	Hispanic/Latino if selected “yes” to “Are you Hispanic or Latino?” Multiracial if selected “no” to “Are you Hispanic or Latino?” and more than one response to “What is your race?” Missing if (1) did not make a selection to “Are you Hispanic or Latino?” or (2) selected “no” to “Are you Hispanic or Latino?” and did not make a selection to “What is your race?” Other categories are based on selected response to “What is your race?” if selected “no” to “Are you Hispanic or Latino?”
		No	
		Missing	
	What is your race? (Select one or more responses)	American Indian or Alaska Native	
		Asian	
		Black or African American	
		Native Hawaiian or Other Pacific Islander	
		White	
		Missing	
Perception of weight	How do you describe your weight?	Very underweight	Very underweight
		Slightly underweight	Slightly underweight
		About the right weight	About the right weight
		Slightly overweight	Slightly overweight
		Very overweight	Very overweight
		Missing	—
Life stressors			
Unstable housing (past 30 days)	During the past 30 days, where did you usually sleep?	In the home of a friend, family member, or other person because I had to leave my home or my parent or guardian cannot afford housing	Yes
		In a shelter or emergency housing	Yes
		In a motel or hotel	Yes
		In a car, park, campground, or other public place	Yes
		I do not have a usual place to sleep	Yes
		In my parent's or guardian's home	No
		Somewhere else	No
		Missing	—
Household adult trying hard to meet basic needs (lifetime)	During your life, how often has there been an adult in your household who tried hard to make sure your basic needs were met, such as looking after your safety and making sure you had clean clothes and enough to eat?	Never	No
		Rarely	No
		Sometimes	No
		Most of the time	Yes
		Always	Yes
		Missing	—
Bullied at school (past 12 months)	During the past 12 months, have you ever been bullied on school property?	Yes	Yes
		No	No
		Missing	—
Missed school due to safety concerns (past 30 days)	During the past 30 days, on how many days did you not go to school because you felt you would be unsafe at school or on your way to or from school?	0 days	No
		1 day	Yes
		2 or 3 days	Yes
		4 or 5 days	Yes
		6 or more days	Yes
		Missing	—
Unfairly disciplined at school (past 12 months)	During the past 12 months, have you been unfairly disciplined at school?	Yes	Yes
		No	No
		Missing	—
Feel close to people at school	Do you agree or disagree that you feel close to people at your school?	Strongly agree	Yes
		Agree	Yes
		Not sure	No
		Disagree	No
		Strongly disagree	No
		Missing	—
Health behaviors			
Met guideline for daily physical activity (past 7 days)	During the past 7 days, on how many days were you physically active for a total of at least 60 min per day?	0 days	No
		1 day	No
		2 days	No
		3 days	No
		4 days	No
		5 days	No
		6 days	No
		7 days	Yes
		Missing	—
Dental visit (past 12 months)	When was the last time you saw a dentist for a check‐up, exam, teeth cleaning, or other dental work?	During the past 12 months	Yes
		Between 12 and 24 months ago	No
		More than 24 months ago	No
		Never	No
		Not Sure	No
		Missing	—
Sleep duration (average school night)	On an average school night, how many hours of sleep do you get?	4 or less hours	< 8 h
		5 h	< 8 h
		6 h	< 8 h
		7 h	< 8 h
		8 h	≥ 8 h
		9 h	≥ 8 h
		10 or more hours	≥ 8 h
		Missing	—

Abbreviation: NH, non‐Hispanic.

Independent variables of interest included two household stressors (unstable housing and sometimes/rarely/never having a household adult trying hard to meet basic needs) and four school stressors (bullied at school, missed school due to safety concerns, unfairly disciplined at school, and not feeling close to others at school [i.e., low school connectedness]). We assessed for potential collinearity among the independent variables.

Four student characteristics and three health behaviors were considered potential confounders as they are often associated with health behaviors and health outcomes. The student characteristics included were: school grade (9th, 10th, 11th, 12th), sex (female, male), race and ethnicity (non‐Hispanic [NH] American Indian/Alaska Native, NH Asian, NH Black or African American, NH Native Hawaiian/Other Pacific Islander, NH White, Hispanic/Latino, and NH multiracial), and perception of weight (very underweight, slightly underweight, about the right weight, slightly overweight, very overweight). Perception of weight was significantly associated (*p* < 0.0001) with body mass index (BMI) based on student‐reported height and weight. We included perception of weight because it might influence nutrition behaviors among some adolescents [22]. The three health behaviors considered were: meeting national guidelines for daily physical activity [23], having a recent dental visit, and sleep duration on an average school night.

The analytic sample size for daily consumption was 12,308 US high school students, which excludes those who were missing data for soda or sports drink consumption and did not indicate they had consumed the beverages at least once per day (missing *n* = 7795). Adjusted analyses for daily consumption further excluded those missing data for grade (*n* = 99), sex (*n* = 56), race and ethnicity (*n* = 190), or weight perception (*n* = 1298). The analytic sample size for heavy daily consumption was 11,980, which excluded those who were missing data for soda or sports drink consumption and did not indicate they had consumed the beverages at least three times per day (missing *n* = 8123). Adjusted analyses for heavy daily consumption further excluded those missing data for grade (*n* = 95), sex (*n* = 53), race and ethnicity (*n* = 182), or weight perception (*n* = 1097). For our sensitivity analysis, the analytic sample size was 11,847 for both daily and heavy daily consumption (missing *n* = 8256). Adjusted sensitivity analyses further excluded those missing data for grade (*n* = 91), sex (*n* = 50), race and ethnicity (*n* = 181), or weight perception (*n* = 1018).

### 2.3 Procedure

The 2023 YRBS was administered at school during one class period; data were collected using tablets programmed with the questionnaire [20]. Additional information regarding the survey plan and methods is described in more detail elsewhere [20].

### 2.4 Data Analysis

We calculated weighted prevalence estimates for daily and heavy daily consumption overall and by student characteristics, life stressors, and health behaviors. We used chi‐square tests to test associations between each of the independent variables of interest and our two outcomes. We calculated unadjusted bivariable prevalence ratios (PR) to compare the prevalence of consumption between students experiencing the life stressor to those who were not. Additionally, we calculated prevalence ratios adjusted (aPR) for student grade, sex, race and ethnicity, and weight perception. To assess for confounding, we also ran models including the three health behaviors (meeting physical activity recommendations, recent dental visit, and sleep duration). While the beta coefficients for the health behaviors were statistically significant in some instances, including these variables as confounders resulted in minimal changes in the PR and did not change the conclusion, so those results are not presented.

We used SAS (v9.4; SAS Institute Inc.; Cary, NC) and SAS‐callable SUDAAN (release 11.0.4; RTI International; Research Triangle Park, NC) for all data management and statistical analyses. Analyses accounted for the complex sample design of the YRBS. We considered chi‐square test results to be statistically significant if *p* < 0.05. PR and aPR were considered statistically significant if the 95% confidence interval (CI) did not cross the null value of 1.0.

## 3 Results

### 3.1 Daily Consumption

Overall, 34.2% (95% CI = 31.0%, 37.5%) of high school students reported consuming soda/sports drink at least once per day (Table 2); results from our sensitivity analysis were comparable (32.1% [95% CI = 29.3, 35.0] of high school students, data not shown). There was a statistically significant association between daily consumption and student grade (*p* = 0.0244), sex (*p* < 0.0001), and perception of weight (*p* < 0.0001) (Table 2). By grade, prevalence of daily consumption ranged from 31.0% (95% CI = 27.1%, 35.3%) among 12th graders to 37.9% (95% CI = 34.0%, 41.9%) among 9th graders. The prevalence of daily consumption was 40.5% (95% CI = 37.3%, 43.9%) among male students and 27.4% (95% CI = 24.0%, 31.1%) among female students. By perceived weight category, prevalence of daily consumption ranged from 30.3% (95% CI = 26.8%, 34.0%) among those who perceived themselves as slightly overweight to 50.7% (95% CI = 41.6%, 59.8%) among those who perceived themselves as very underweight. Although there were not statistically significant differences overall by race and ethnicity (*p* = 0.1629), the prevalence of daily consumption ranged from 18.3% (95% CI = 11.7%, 27.6%) among Asian students to 40.3% (95% CI = 32.9%, 48.2%) among Black or African American students (Table 2).

**TABLE 2:** Unweighted counts, weighted prevalence estimates and associated 95% confidence intervals for soda and sports drink consumption by student characteristics, life stressors, and health behaviors—Youth Risk Behavior Survey, High School Students, United States, 2023.

	Daily soda/sports drink consumption (≥ 1 time per day, past 7 days)			Heavy daily soda/sports drink consumption (≥ 3 times per day, past 7 days)		
	*n*	Prevalence (95% CI)	*p*	*n*	Prevalence (95% CI)	*p*
Overall	12,308	34.2 (31.0, 37.5)	—	11,980	7.9 (6.4, 9.6)	—
Missing	7795	—		8123	—	
Student characteristics						
School grade level	12,209	—	0.0244*	11,885	—	0.0290*
9th	3243	37.9 (34.0, 41.9)		3162	9.5 (7.5, 12.1)	
10th	3268	34.4 (30.5, 38.5)		3168	7.9 (6.0, 10.4)	
11th	2904	33.4 (29.5, 37.5)		2817	7.6 (5.9, 9.7)	
12th	2794	31.0 (27.1, 35.3)		2738	6.0 (4.4, 8.2)	
Missing	99	—		95	—	
Sex	12,240	—	< 0.0001*	11,915	—	0.0001*
Female	6054	27.4 (24.0, 31.1)		5900	6.0 (4.6, 7.8)	
Male	6186	40.5 (37.3, 43.9)		6015	9.6 (7.9, 11.7)	
Missing	68	—		65	—	
Race and ethnicity	12,079	—	0.1629	11,760	—	0.0309*
American Indian/Alaska Native, NH	1135	35.0 (22.0, 50.6)		1117	12.7 (4.5, 30.7)^a^	
Asian, NH	470	18.3 (11.7, 27.6)		457	5.5 (3.0, 9.9)^a^	
Black/African American, NH	1308	40.3 (32.9, 48.2)		1290	14.3 (10.2, 19.7)	
Hispanic/Latino	2500	33.7 (30.3, 37.3)		2437	7.6 (6.0, 9.5)	
Multirace, NH	1286	33.9 (26.5, 42.1)		1256	8.0 (5.2, 12.1)	
Native Hawaiian/Other Pacific Islander, NH	73	39.7 (21.4, 61.5)		68	15.1 (3.7, 45.4)^a^	
White, NH	5307	34.6 (31.2, 38.2)		5135	6.4 (4.8, 8.4)	
Missing	229	—		220	—	
Perception of weight	10,927	—	< 0.0001*	10,808	—	0.0002*
Very underweight	349	50.7 (41.6, 59.8)		341	15.5 (10.1, 22.9)	
Slightly underweight	1738	40.1 (35.2, 45.1)		1722	9.5 (7.2, 12.4)	
About right	5187	31.3 (28.2, 34.4)		5130	6.3 (5.1, 7.9)	
Slightly overweight	2943	30.3 (26.8, 34.0)		2915	5.9 (4.4, 7.9)	
Very overweight	710	36.7 (30.8, 42.9)		700	12.2 (8.7, 16.8)	
Missing	1381	—		1172	—	
Life stressors						
Unstable housing (past 30 days)	11,127	—	0.0024*	10,808	—	0.0005*
Yes	326	58.2 (43.2, 71.7)		293	25.4 (16.5, 36.9)	
No	10,801	33.6 (30.5, 36.9)		10,515	7.3 (5.9, 9.0)	
Missing	1181	—		1172	—	
Household adult trying hard to meet basic needs (lifetime)	10,974	—	0.0005*	10,733	—	0.0021*
Yes	9486	32.2 (28.9, 35.5)		9280	7.0 (5.6, 8.6)	
No	1488	42.5 (37.9, 47.2)		1453	10.9 (8.2, 14.4)	
Missing	1334	—		1247	—	
Bullied at school (past 12 months)	12,189	—	0.0001*	11,868	—	0.0011*
Yes	2524	41.9 (37.0, 46.9)		2444	11.5 (8.7, 15.2)	
No	9665	32.4 (29.4, 35.6)		9424	6.9 (5.6, 8.5)	
Missing	119	—		112	—	
Missed school due to safety concerns (past 30 days)	11,309	—	0.0008*	10,985	—	0.0008*
Yes	1554	41.7 (35.6, 48.1)		1507	12.4 (8.8, 17.1)	
No	9755	33.1 (29.9, 36.5)		9478	7.2 (5.8, 8.8)	
Missing	999	—		995	—	
Unfairly disciplined at school (past 12 months)	10,470	—	0.0007*	10,455	—	0.0002*
Yes	2142	39.3 (33.8, 45.0)		2140	11.2 (8.7, 14.3)	
No	8328	30.9 (28.2, 33.6)		8315	6.3 (5.1, 7.7)	
Missing	1838	—		1525	—	
Feel close to people at school	10,592	—	0.0914	10,520	—	0.2221
Yes	5711	33.8 (30.5, 37.3)		5677	7.7 (6.1, 9.6)	
No	4881	31.6 (28.6, 34.7)		4843	6.8 (5.3, 8.6)	
Missing	1716			1460	—	
Health behaviors						
Met guideline for daily physical activity (past 7 days)	12,096	—	0.0001*	11,772	—	0.0011*
Yes	3139	43.6 (39.8, 47.4)		3064	10.1 (8.4, 12.1)	
No	8957	31.4 (27.9, 35.0)		8708	7.1 (5.6, 8.9)	
Missing	212	—		208	—	
Dental visit (past 12 months)	12,076	—	0.0002*	11,759	—	0.0008*
Yes	8935	32.7 (29.5, 36.0)		8725	6.6 (5.2, 8.4)	
No	3141	39.2 (35.1, 43.5)		3034	11.3 (8.9, 14.2)	
Missing	232	—		221	—	
Sleep duration (average school night)	11,027	—	0.9247	10,723	—	0.3302
≥ 8 h	2507	34.4 (30.2, 38.8)		2442	7.1 (5.2, 9.6)	
< 8 h	8520	34.2 (31.0, 37.6)		8281	7.9 (6.5, 9.7)	
Missing	1281	—		1257	—	

Abbreviations: CI, confidence interval; NH, non‐Hispanic.

^a^ Relative standard error for estimate is > 30%. Estimate should be interpreted with caution.

* *p* < 0.05.

The prevalence of daily consumption was 31.4% (95% CI = 27.9%, 35.0%) among those not meeting daily physical activity guidelines compared with 43.6% (95% CI = 39.8%, 47.4%) among those who did (*p* = 0.0001). The prevalence of daily consumption was lower among students who reported having a dental visit in the past 12 months (32.7%, 95% CI = 29.5%, 36.0%) compared with 39.2% (95% CI = 35.1%, 43.5%) among those who did not (*p* = 0.0002). There was no statistically significant association between daily consumption and sleep duration (*p* = 0.9247; ≥ 8 h 34.4% [95% CI = 30.2%, 38.8%]; < 8 h: 34.2% [95% CI = 31.0%, 37.6%]).

**FIGURE 1:**
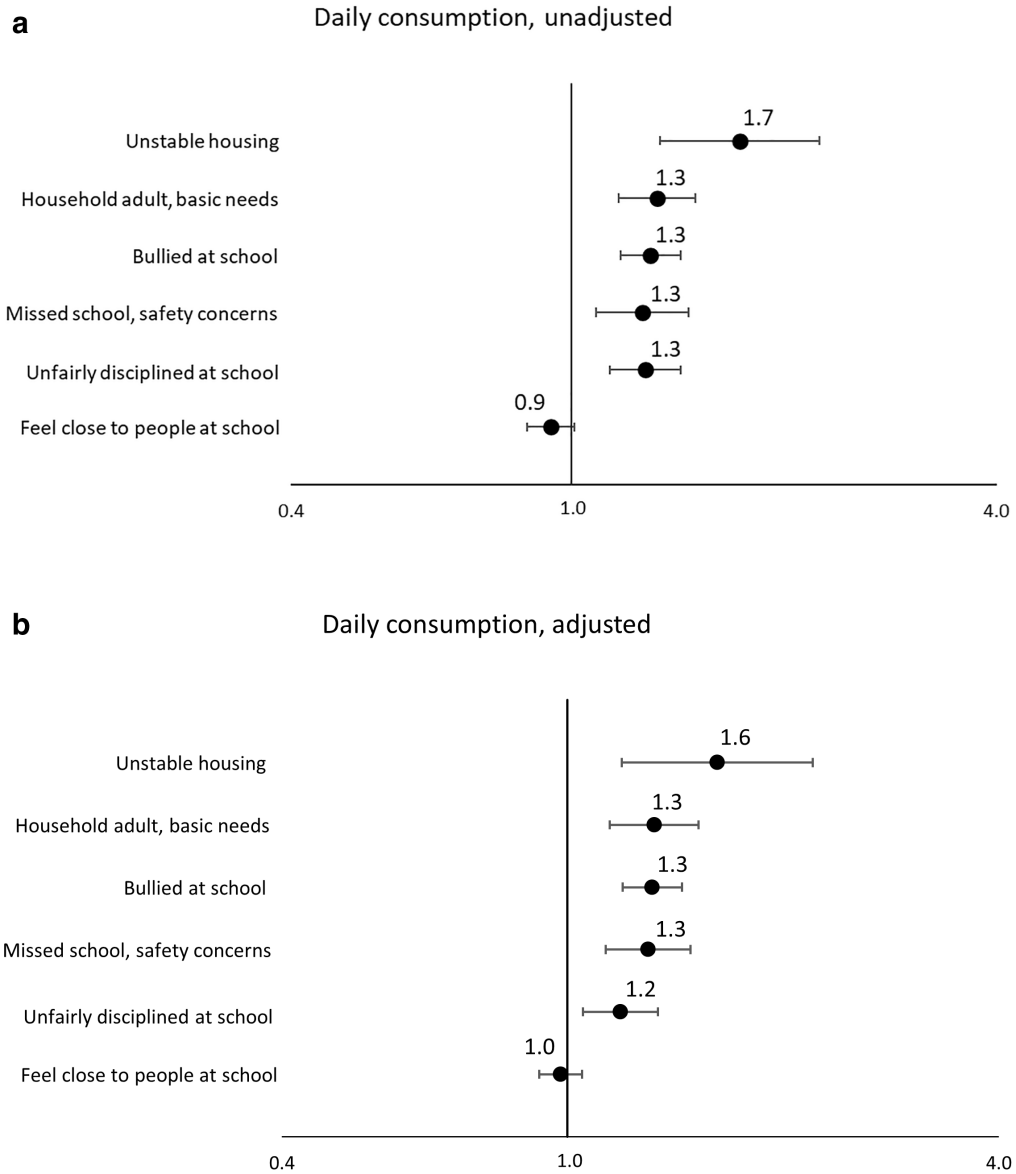
(a) Unadjusted prevalence ratios^a^ of daily (≥ 1 time/day) soda/pop/sports drink consumption by life stressors on log scale—Youth Risk Behavior Survey, High School Students, United States, 2023. ^a^Models excluded observations with missing data for soda/pop or sports drink consumption if sum was less than 1×/day (*n* = 7795) and the respective independent variable: unstable housing (past 30 days; *n* = 1181), household adult trying hard to meet basic needs (lifetime; *n* = 1334), bullied at school (past 12 months; *n* = 119), missed school due to safety concerns (past 30 days; *n* = 999), unfairly disciplined at school (past 12 months; *n* = 1838), feel close to people at school (*n* = 1716). The referent group for each model was the group not experiencing the stressor. The number of observations included in models were as follows: unstable housing (*n* = 11,127), household adult trying hard to meet basic needs (*n* = 10,974), bullied at school (*n* = 12,189), missed school due to safety concerns (*n* = 11,309), unfairly disciplined at school (*n* = 10,470), feel close to people at school (*n* = 10,592). (b) Prevalence ratios of daily (≥ 1 time/day) soda/pop/sports drink consumption by life stressors adjusted for student characteristics^a^ on log scale—Youth Risk Behavior Survey, High School Students, United States, 2023. ^a^Models excluded observations with missing data for soda/pop or sports drink consumption if sum was less than 1×/day (*n* = 7795), student grade (*n* = 99), sex (*n* = 56), race and ethnicity (*n* = 190), weight perception (*n* = 1298), or the respective independent variable: unstable housing (past 30 days; *n* = 198), household adult trying hard to meet basic needs (lifetime; *n* = 283), bullied at school (past 12 months; *n* = 65), missed school due to safety concerns (past 30 days; *n* = 39), unfairly disciplined at school (past 12 months; *n* = 457), feel close to people at school (*n* = 346). The referent group for each model was the group not experiencing the stressor. The number of observations included in models was as follows: unstable housing (*n* = 10,467), household adult trying hard to meet basic needs (*n* = 10,382), bullied at school (*n* = 10,600), missed school due to safety concerns (*n* = 10,626), unfairly disciplined at school (*n* = 10,208), feel close to people at school (*n* = 10,319).

Except for low school connectedness (*p* = 0.0914), there was a statistically significant association between life stressors and daily consumption of soda/sports drink (*p* < 0.05); those experiencing the stressors had a higher prevalence of consumption than those not experiencing the stressors. The unadjusted (Figure 1a) and adjusted (Figure 1b) results were similar.

The prevalence of daily consumption was higher among those who experienced unstable housing (58.2%, 95% CI = 43.2%, 71.7%) compared with those who had not (33.6% [95% CI = 30.5%, 36.9%]; aPR: 1.6 [95% CI = 1.2, 2.2]) (Table 2 and Figure 1b). The prevalence of daily consumption among those who sometimes/rarely/never had a household adult trying hard to meet their basic needs was 42.5% (95% CI = 37.9%, 47.2%) compared with 32.2% (95% CI = 28.9%, 35.5%) among those who did (aPR: 1.3 [95% CI = 1.1, 1.5]). The prevalence of daily consumption was higher among those who had been unfairly disciplined at school (39.3%, [95% CI = 33.8%, 45.0%]) compared with those who had not (30.9%, [95% CI = 28.2%, 33.6%]; aPR: 1.2 [95% CI = 1.1, 1.3]). Those who did not feel close to others at school reported a daily consumption prevalence of 31.6% (95% CI = 28.6%, 34.7%) versus 33.8% (95% CI = 30.5%, 37.3%) among those who did (aPR: 1.0 [95% CI = 0.9, 1.0]).

Results from the sensitivity analysis were generally similar. The prevalence of daily soda/sports drink consumption was statistically significantly higher among those sometimes/rarely/never having a household adult trying hard to meet their basic needs (aPR = 1.3 [95% CI = 1.1, 1.5]), bullied at school (aPR: 1.3 [95% CI = 1.2, 1.4]), and missing school due to safety concerns (aPR: 1.3 [95% CI = 1.1, 1.4]) compared to students not experiencing those stressors. There was no statistically significant difference in prevalence of daily consumption among those experiencing unstable housing (aPR: 1.5 [95% CI = 1.0, 2.1]), being unfairly disciplined at school (aPR: 1.2 [95% CI = 1.0, 1.3]), or not feeling close to others at school (aPR: 1.0 [95% CI = 0.9, 1.0]).

### 3.2 Heavy Daily Consumption

The overall prevalence of heavy daily consumption (≥ 3 times per day) among high school students was 7.9% (95% CI = 6.4%, 9.6%) (Table 2); our sensitivity analysis results were comparable (7.0% [95% CI = 5.7, 8.6] of high school students, data not shown). Conclusions based on tests for associations between heavy daily consumption and student characteristics, life stressors, and health behaviors were similar to results for daily consumption, although there was a statistically significant association between student race and ethnicity and heavy daily consumption (*p* = 0.0309) (Table 2). The prevalence of heavy daily consumption ranged from about 6% (NH Asian: 5.5% [95% CI = 3.0%, 9.9%], NH White: 6.4% [95% CI = 4.8%, 8.4%]) to 14.3% (95% CI = 10.2%, 19.7%) among NH Black or African American students and 15.1% (95% CI = 3.7%, 45.4%) among NH Native Hawaiian/Other Pacific Islander students.

**FIGURE 2:**
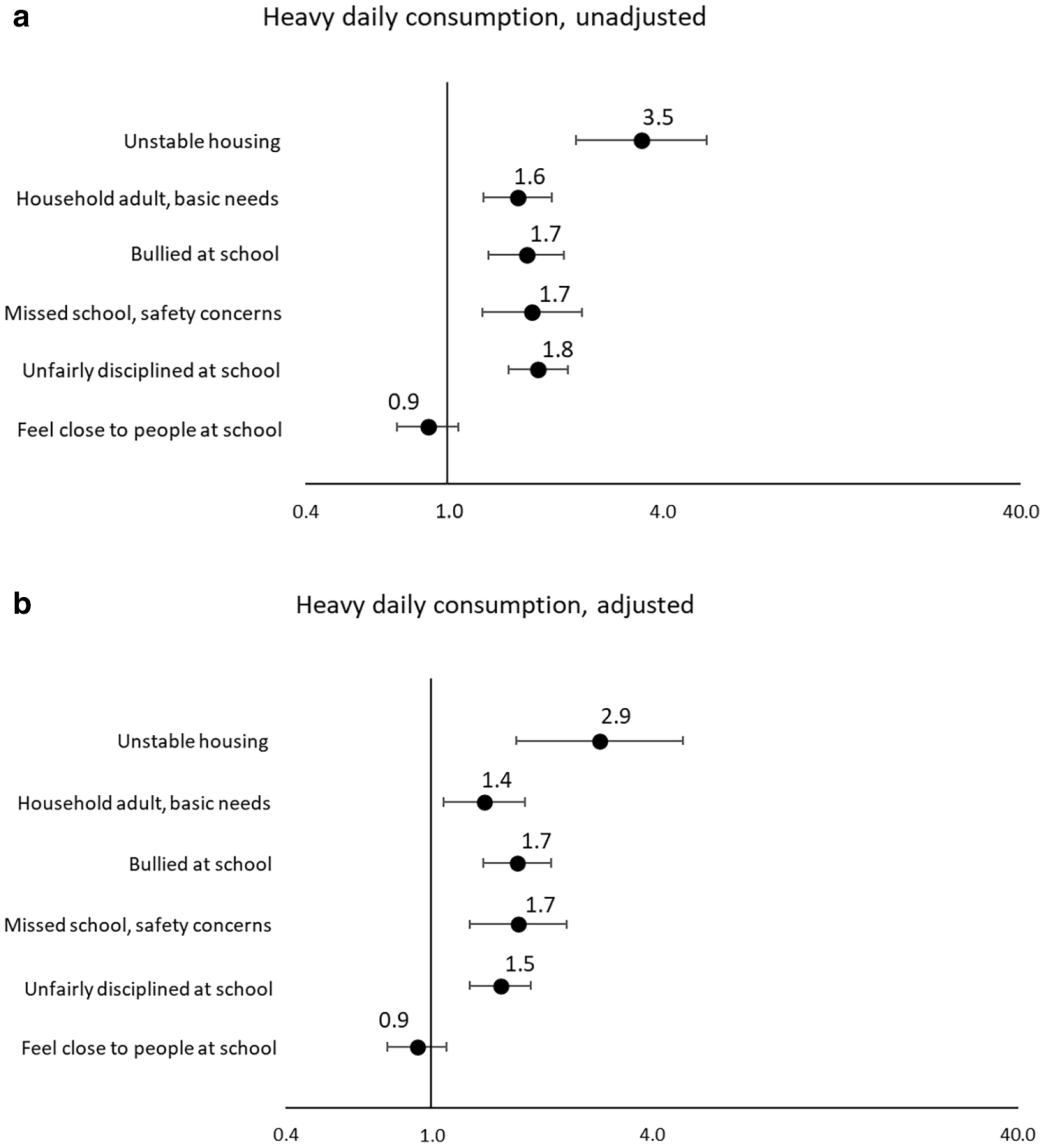
(a) Unadjusted prevalence ratios^a^ of heavy daily (≥ 3 times/day) soda/pop/sports drink consumption by life stressors on log scale—Youth Risk Behavior Survey, High School Students, United States, 2023. ^a^Models excluded observations with missing data for soda/pop or sports drink consumption if sum was less than 3×/day (*n* = 8123) and the respective independent variable: unstable housing (past 30 days; *n* = 1172), household adult trying hard to meet basic needs (lifetime; *n* = 1247), bullied at school (past 12 months; *n* = 112), missed school due to safety concerns (past 30 days; *n* = 995), unfairly disciplined at school (past 12 months; *n* = 1525), feel close to people at school (*n* = 1460). The referent group for each model was the group not experiencing the stressor. The number of observations included in models were as follows: unstable housing (*n* = 10,808), household adult trying hard to meet basic needs (*n* = 10,733), bullied at school (*n* = 11,868), missed school due to safety concerns (*n* = 10,985), unfairly disciplined at school (*n* = 10,455), feel close to people at school (*n* = 10,520). (b) Prevalence ratios of heavy daily (≥ 3 time/day) soda/pop/sports drink consumption by life stressors adjusted for student characteristics^a^ on log scale—Youth Risk Behavior Survey, High School Students, United States, 2023. ^a^Models excluded observations with missing data for soda/pop or sports drink consumption if sum was less than 3×/day (*n* = 8123), student grade (*n* = 95), sex (*n* = 53), race and ethnicity (*n* = 182), weight perception (*n* = 1097), or the respective independent variable: unstable housing (past 30 days; *n* = 195), household adult trying hard to meet basic needs (lifetime; *n* = 228), bullied at school (past 12 months; *n* = 61), missed school due to safety concerns (past 30 days; *n* = 37), unfairly disciplined at school (past 12 months; *n* = 359), feel close to people at school (*n* = 301). The referent group for each model was the group not experiencing the stressor. The number of observations included in models was as follows: unstable housing (*n* = 10,358), household adult trying hard to meet basic needs (*n* = 10,325), bullied at school (*n* = 10,492), missed school due to safety concerns (*n* = 10,516), unfairly disciplined at school (*n* = 10,194), feel close to people at school (*n* = 10,252).

The unadjusted PR are presented in Figure 2a, and there was little difference in the aPR (Figure 2b). Similar to daily consumption, heavy daily consumption was higher among students who experienced life stressors versus those who did not. For example, the prevalence of heavy daily consumption among those who experienced unstable housing was 25.4% (95% CI = 16.5%, 36.9%) compared with 7.3% (95% CI = 5.9%, 9.0%) among those who had not (aPR = 2.9 [95% CI = 1.7, 4.9]) (Table 2, Figure 2b). The prevalence of heavy daily consumption was higher among those who experienced bullying at school (11.5%, [95% CI = 8.7%, 15.2%]) versus those who had not (6.9% [95% CI = 5.6%, 8.5%]; aPR: 1.7 [95% CI = 1.4, 2.1]). Among students who missed school due to safety concerns, 12.4% (95% CI = 8.8%, 17.1%) were heavy daily consumers versus 7.2% (95% CI = 5.8%, 8.8%) of those who had not missed school due to safety concerns (aPR = 1.7 [95% CI = 1.3, 2.3]). However, there was no statistically significant association between low school connectedness (*p* = 0.2221) and heavy daily consumption (aPR = 0.9 [95% CI = 0.8, 1.1]).

Results from the sensitivity analysis for heavy daily consumption were also generally similar. The prevalence was statistically significantly higher among those experiencing unstable housing (aPR = 2.5 [95% CI = 1.4, 4.4]), being bullied at school (aPR = 1.7 [95% CI = 1.3, 2.1]), missing school due to safety concerns (aPR = 1.6 [95% CI = 1.2, 2.2]), and being unfairly disciplined at school (aPR = 1.5 [95% CI = 1.2, 1.8]) compared to students not experiencing those stressors. There was no statistically significant difference in prevalence of heavy daily consumption among those sometimes/rarely/never having a household adult trying hard to meet their basic needs (aPR = 1.3 [95% CI = 1.0, 1.7]) and not feeling close to others at school (aPR = 0.9 [95% CI = 0.7, 1.1]).

## 4 Discussion

Among a nationally representative sample of more than 10,000 US high school students in 2023, the prevalences of daily and heavy daily consumption of soda/sports drink were 34.2% and 7.9%, respectively. The prevalence of daily consumption was 20%–60% higher among high school students who reported experiencing life stressors, including unstable housing, lacking a household adult trying to meet basic needs, being bullied at school, missing school due to safety concerns, and feeling unfairly disciplined at school—compared with students who did not experience those stressors. To our knowledge, this is the first study among US high school students to report the associations of these life stressors and soda and sports drink consumption. These findings contribute to the body of literature demonstrating that life stressors can influence health behaviors among adolescents [13, 16].

While the reason for these associations is unclear, youth might use SSB consumption as a way to lessen negative feelings associated with stress [12, 16, 17]. Contextual factors, such as physical, cognitive, and emotional health [24], are important considerations as these factors might influence individual SSB consumption behavior. Park et al. hypothesized that some adolescents who have been victimized (e.g., bullied at school) might consume sugary items (e.g., soda/pop) as a coping mechanism [18]. Also, other results show that students with relatively high stress levels consume a lower quality diet [25] and greater amounts of sugar [26] compared to students with lower stress levels. Students facing life stressors might need additional strategies to support healthy behaviors, including nutrition.

Additionally, SSB consumption prevalence might be higher among some adolescents due to differences in exposure and availability. For example, prior research indicates that unhealthy foods and beverages are disproportionately marketed in low‐income neighborhoods [27] and that the price of healthy food could be twice as high as unhealthy food [28]. These factors might be particularly important considerations for adolescents who reported experiencing household stressors.

While some adolescents might consume SSBs more frequently to cope with stressors or as a reflection of marketing and availability, for others, SSB consumption might be a precursor to stress. For instance, prior research indicates a positive association between consumption of SSBs and other sugary foods and hyperactivity [29, 30]. Thus, students who consume SSBs and experience hyperactivity might also report feeling unfairly disciplined at school.

Our finding of no statistically significant association between feeling connected to others at school and the prevalence of soda/sports drink consumption might reflect a lower degree of influence of this stressor on beverage consumption compared with other assessed stressors. However, studies show that student SSB consumption behaviors might be modeled after observed teachers' SSB consumption [31] or perceived consumption behavior of peers [32]. In other research, adolescents have reported positive perceptions of SSB consumption in conjunction with special social gatherings [33], which might lead to a higher prevalence of consumption.

Life stressors, like those included in this analysis, can occur in both school and household environments. These environments also offer opportunities to intervene to support well‐being among youth and mitigate the negative effects of stressors. The school, home, and wider environments have the potential to positively or negatively shape behaviors, including nutrition and coping behaviors, during this critical developmental life phase.

### 4.1 Implications for School Health Policy, Practice, and Equity

This description of associations between soda and sports drink consumption and life stressors among US high school students might help school administrators and public health practitioners create or expand focused programs and interventions to reduce soda and sports drink consumption. Comprehensive strategies that address both stressors and health behaviors, including SSB consumption, might be most beneficial.

Schools can support students by implementing policies, practices, and programs aligned with the Whole School, Whole Community, Whole Child (WSCC) framework [34]. This student‐centered framework comprises 10 components including counseling, psychological and social services; nutrition environment and services; social and emotional climate, and physical environment. Schools can use the WSCC framework to address both health behaviors and underlying stressors that influence well‐being. For example, schools might be able to provide a range of services related to mental, behavioral, social, and emotional health including conducting assessments, offering counseling and consultation, and referring students and their families to school and community support services, which could include assistance with housing and food [34]. Schools can reduce stress and minimize bullying by establishing safe physical environments and a positive social and emotional climate. By adopting the whole child approach of the WSCC, schools can help students establish behaviors that could have long‐lasting positive health effects. These comprehensive strategies might be particularly helpful in supporting healthy behaviors (e.g., reduced SSB consumption) among students experiencing life stressors, as our results show these students had a relatively high prevalence of soda/sports drink consumption.

Comprehensive wellness strategies can be complemented by school‐based policies and environmental factors that might also help reduce SSB consumption by addressing availability, cost, and marketing [35]. For example, universal free meals have been shown to reduce food insufficiency among youth [36] and Smart Snacks in School establish nutrition standards for foods and beverages sold during the school day [37]. However, it is important to recognize that adoption and implementation of policies and interventions vary greatly across districts and schools [38, 39, 40]. Comprehensive approaches addressing student health like the WSCC framework might help bolster the sense of community among peers, foster a healthier school environment, and reinforce school initiatives to improve nutrition [34].

Some household factors including household income, beverage availability in the household, and parental behaviors, have also been shown to influence beverage choices. Household income has previously been associated with both life stressors (e.g., housing instability) [41] and SSB consumption [42]. For adolescents experiencing certain life stressors such as unstable housing or lacking a household adult trying to meet basic needs, addressing these overarching aspects of well‐being likely would take priority over nutrition behavior modification. For students not experiencing paramount life stressors such as those, there are a variety of evidence‐based interventions that can be implemented in the household to foster healthy behaviors among adolescents and reduce SSB consumption. For example, SSB availability in the home [43] and parental consumption of SSBs have been associated with youth SSB intake [44, 45, 46]. Some families might benefit from nutrition education and behavior modification programs (e.g., Smart Moves for Kids) [47] that encourage healthy nutrition and physical activity behaviors. However, to better meet the needs of adolescents experiencing life stressors, evidence‐based interventions might be adapted to emphasize healthy coping strategies in the household environment.

Additional research to better understand the relationship between life stressors and SSB consumption among US high school students could further guide strategies in both the school and household environments.

## 5 Limitations

This study had notable strengths. First, we used data from a nationally representative sample of US high school students in both public and private schools. Additionally, we analyzed multiple stressor variables, some of which were included in the 2023 national YRBS for the first time. This study also had limitations. First, several respondents had missing data for our variables of interest. Some schools might have been selected to participate in the national sample as well as a state or large urban school district sample. In these instances, the students responded to only one survey, which might have been a state or large urban school district survey that excluded some of the questions (e.g., sports drink consumption) on the national survey. Missing data might also result from the student not answering the question or the response being set to missing based on an improbable response. Unfortunately, based on available information, we are unable to determine which reason is applicable for each of the missing responses [20]. We are also unable to determine how the extent of missingness might have influenced our results. But there were differences in the distribution of student grade, race and ethnicity, and weight perception among those who were missing data for our outcomes of interest versus those who were not (Table S1). For example, those who had missing outcome data—compared to those who were not missing outcome data—were more likely to be in younger grades, NH White, NH Asian, or perceive themselves as very underweight. Second, we are unable to determine the degree to which biases might have influenced underreporting or overreporting of soda/sports drink consumption and life experiences. A different self‐reported beverage consumption questionnaire generated overestimates compared to 24‐h dietary recall among adolescents, but the differences were small and might not be meaningful in practice [48]. Furthermore, many YRBS questions have been shown to be reliable [49]. Third, students not in school on the day of survey administration and those who are homeschooled are not represented. Students experiencing specific life stressors, such as unstable housing, might experience more school absenteeism than those in stable housing environments. This might have attenuated our results. Fourth, since the YRBS is a cross‐sectional survey, causation nor directionality of associations can be determined. Fifth, the soda and sports drink consumption data in YRBS are based on frequency of consumption rather than amount; these data cannot be used to calculate the amount of sugar consumed via beverages. Sixth, soda and sports drink (excluding diet and low‐calorie versions) were the only SSBs included in the 2023 YRBS, although many other SSBs are available (e.g., sugar‐sweetened coffees or teas, and energy drinks). Results might differ if a broader range of SSBs, including those with higher caffeine content, were included. Future research could explore the types of SSBs consumed by this population and their association with life stressors. Additionally, each of the stressors included in this study might vary in severity, duration, and influence on health and well‐being. We were unable to incorporate these aspects of life stressors in our analyses since these details were not captured in the YRBS. Also, students might experience multiple concurrent stressors. Future research could explore the mechanisms driving the association between experiencing these life stressors and SSB consumption—to develop more meaningful interventions.

## 6 Conclusion

The prevalence of daily and heavy daily consumption of soda/sports drink was higher among US high school students who experienced life stressors compared with those who did not. Comprehensive wellness strategies that support adolescents experiencing stressors in their household and school environments might help adolescents develop healthier behaviors, such as reducing SSB consumption, and improve current and future overall health and well‐being.

## Funding

The authors have nothing to report.

## Disclosure

The findings and conclusions in this manuscript are those of the authors and do not necessarily represent the official position of the Centers for Disease Control and Prevention.

## Ethics Statement

The authors have nothing to report.

## Conflicts of Interest

The authors declare no conflicts of interest.

## Supporting information


**Table S1:** Distribution of student characteristics by outcome variable status—Youth Risk Behavior Survey, High School Students, United States, 2023.

## Data Availability

The data that support the findings of this study are openly available in YRBS Data and Documentation at https://www.cdc.gov/yrbs/data/index.html.
