# Supplementary material for: Soda and Sports Drink Consumption and Life Stressors Among High School Students: Youth Risk Behavior Survey, 2023
Source: J Sch Health. 2026 Aug 4;96(8):e70201. doi: 10.1111/josh.70201 (PMC13438121; doi:10.1111/josh.70201)
Supplement: Supplementary file 1 [file JOSH-96-0-s001.docx]

**Supplemental Table.** Distribution of student characteristics by outcome variable status — Youth Risk Behavior Survey, High School Students, United States, 2023

|  | **Daily soda/sports drink consumption** | | | | | | | | **Heavy daily soda/sports drink consumption** | | | | | | | |
| --- | --- | --- | --- | --- | --- | --- | --- | --- | --- | --- | --- | --- | --- | --- | --- | --- |
|  | **(≥ 1 time per day, past 7 days)** | | | | | | | | **(≥ 3 times per day, past 7 days)** | | | | | | | |
|  | **Outcome data** | | | **No outcome data** | | | **Wgt** | **Unwgt** | **Outcome data** | | | **No outcome data** | | | **Wgt** | **Unwgt** |
| **Student characteristics** | ***Unwgt n*** | **Wgt %** | **Unwgt %** | ***Unwgt n*** | **Wgt %** | **Unwgt %** | ***p*-value** | ***p*-value** | ***Unwgt n*** | **Wgt %** | **Unwgt %** | ***Unwgt n*** | **Wgt %** | **Unwgt %** | ***p*-value** | ***p*-value** |
| **School grade level** |  |  |  |  |  |  | 0.0879 | <.0001 |  |  |  |  |  |  | 0.1391 | <.0001 |
| 9th | 3,243 | 26.2 | 26.6 | 2,437 | 27.1 | 31.8 |  |  | 3,162 | 26.4 | 26.6 | 2,518 | 26.6 | 31.6 |  |  |
| 10th | 3,268 | 25.3 | 26.8 | 2,142 | 26.8 | 28.0 |  |  | 3,168 | 25.3 | 26.7 | 2,242 | 26.9 | 28.1 |  |  |
| 11th | 2,904 | 23.5 | 23.8 | 1,907 | 26.1 | 24.9 |  |  | 2,817 | 23.5 | 23.7 | 1,994 | 26.0 | 25.0 |  |  |
| 12th | 2,794 | 25.0 | 22.9 | 1,167 | 20.0 | 15.3 |  |  | 2,738 | 24.8 | 23.0 | 1,223 | 20.5 | 15.3 |  |  |
| **Sex** |  |  |  |  |  |  | 0.7155 | 0.7339 |  |  |  |  |  |  | 0.6924 | 0.8936 |
| Female | 6,054 | 47.9 | 49.5 | 3,830 | 48.5 | 49.7 |  |  | 5,900 | 47.9 | 49.5 | 3,984 | 48.6 | 49.6 |  |  |
| Male | 6,186 | 52.1 | 50.5 | 3,875 | 51.5 | 50.3 |  |  | 6,015 | 52.1 | 50.5 | 4,046 | 51.4 | 50.4 |  |  |
| **Race and ethnicity** |  |  |  |  |  |  | 0.9252 | <.0001 |  |  |  |  |  |  | 0.9455 | <.0001 |
| American Indian/Alaska Native, NH | 1,135 | 0.3 | 9.4 | 199 | 0.4 | 2.6 |  |  | 1,117 | 0.3 | 9.5 | 217 | 0.4 | 2.7 |  |  |
| Asian, NH | 470 | 3.7 | 3.9 | 525 | 5.5 | 6.9 |  |  | 457 | 3.8 | 3.9 | 538 | 5.4 | 6.8 |  |  |
| Black/African American, NH | 1,308 | 13.8 | 10.8 | 483 | 12.2 | 6.3 |  |  | 1,290 | 13.9 | 11.0 | 501 | 12.1 | 6.3 |  |  |
| Hispanic/Latino | 2,500 | 28.6 | 20.7 | 1,494 | 24.8 | 19.5 |  |  | 2,437 | 28.6 | 20.7 | 1,557 | 25.1 | 19.5 |  |  |
| Multi-race, NH | 1,286 | 5.8 | 10.7 | 528 | 6.8 | 6.9 |  |  | 1,256 | 5.9 | 10.7 | 558 | 6.7 | 7.0 |  |  |
| Native Hawaiian/Other Pacific Islander, NH | 73 | 0.3 | 0.6 | 32 | 0.5 | 0.4 |  |  | 68 | 0.3 | 0.6 | 37 | 0.5 | 0.5 |  |  |
| White, NH | 5,307 | 47.3 | 43.9 | 4,393 | 49.9 | 57.4 |  |  | 5,135 | 47.3 | 43.7 | 4,565 | 49.9 | 57.3 |  |  |
| **Perception of weight** |  |  |  |  |  |  | <.0001 | <.0001 |  |  |  |  |  |  | <.0001 | <.0001 |
| Very underweight | 349 | 3.1 | 3.2 | 125 | 21.0 | 14.4 |  |  | 341 | 3.1 | 3.2 | 133 | 19.6 | 13.5 |  |  |
| Slightly underweight | 1,738 | 16.6 | 15.9 | 133 | 18.2 | 15.3 |  |  | 1,722 | 16.6 | 15.9 | 149 | 17.8 | 15.1 |  |  |
| About right | 5,187 | 48.8 | 47.5 | 368 | 37.9 | 42.4 |  |  | 5,130 | 48.9 | 47.5 | 425 | 38.6 | 43.1 |  |  |
| Slightly overweight | 2,943 | 25.5 | 26.9 | 196 | 18.1 | 22.6 |  |  | 2,915 | 25.5 | 27.0 | 224 | 18.8 | 22.7 |  |  |
| Very overweight | 710 | 6.0 | 6.5 | 46 | 4.7 | 5.3 |  |  | 700 | 6.0 | 6.5 | 56 | 5.2 | 5.7 |  |  |
|  |  |  |  |  |  |  |  |  |  |  |  |  |  |  |  |  |
| Unwgt: unweighted | |  |  |  |  |  |  |  |  |  |  |  |  |  |  |  |
| Wgt: weighted |  |  |  |  |  |  |  |  |  |  |  |  |  |  |  |  |
